# Research of a Cross-Interference Suppression Method for Piezoresistive Three-Dimensional Force Sensor

**DOI:** 10.3390/s23094573

**Published:** 2023-05-08

**Authors:** You Zhao, Yulong Zhao

**Affiliations:** State Key Laboratory for Manufacturing Systems Engineering, Xi’an Jiaotong University, Xi’an 710049, China

**Keywords:** three-dimensional, force sensor, cross-interference, suppression, method

## Abstract

Cross-interference is not only an important factor that affects the measuring accuracy of three-dimensional force sensors, but also a technical difficulty in three-dimensional force sensor design. In this paper, a cross-interference suppression method is proposed, based on the octagonal ring’s structural symmetry as well as Wheatstone bridge’s balance principle. Then, three-dimensional force sensors are developed and tested to verify the feasibility of the proposed method. Experimental results show that the proposed method is effective in cross-interference suppression, and the optimal cross-interference error of the developed sensors is 1.03%. By optimizing the positioning error, angle deviation, and bonding process of strain gauges, the cross-interference error of the sensor can be further reduced to −0.36%.

## 1. Introduction

Three-dimensional force (generally expressed as *F_x_*, *F_y_*, and *F_z_*) refers to the forces along the X, Y, and Z directions in the space Cartesian coordinate system. Three-dimensional force measurement is necessary in many important fields, such as precision manufacturing and robots [1,2,3], medical equipment [4,5], wearable devices [6,7], structural health monitoring of large buildings [8], etc. Three-dimensional force sensor is an important tool for measuring three-dimensional forces. However, in practical application, there is often a situation where the sensor is interfered by *F_y_* (or *F_z_*) when measuring *F_x_*, which indicates that the measurement results of *F_x_* include the interference of *F_y_*. Similarly, the measurement results of *F_y_* include the interference of *F_x_*. This is defined as the cross-interference between *F_x_* and *F_y_*. Cross-interference error is an important index related to force measuring accuracy. It is a quantitative indicator representing the interference of *F_y_* on *F_x_*’s measurement result, which reflects the ability of three-dimensional force sensor to measure force in a specific direction without being interfered with forces from other directions. Cross-interference error can be calculated as Equation (1) [9], where *E_Fy_*_→*Fx*_ indicates the cross-interference error of *F_y_* on *F_x_*’s measurement result, *FSO_Fx_* and *FSO_Fy_* represent the full-scale output of *F_x_* measuring result under *F_x_* and *F_y_*, respectively.

Scholars have carried out a large amount of fruitful research on cross-interference error suppression, especially in structural design. For example, the famous piezoelectric force sensor company KISTLER has developed a series of three-dimensional force sensors for different applications, with a typical cross-interference error between ±1% and ±3% for the measuring range of 3~5 kN [10]. Jing Li developed a miniature cross-shaped three-dimensional piezoresistive force sensor [11], with cross-interference errors of different directions in the range of 4%~25%. Zexia He designed a six-axis force sensor based on a 3D capacitor structure with a cross-shape configuration of the shear force sensing cell [12]. The maximum interference errors for *F_x_*, *F_y_*, *F_z_*, *M_x_*, *M_y_*, and *M_z_* directions are 1.95%, 2.01%, 1.58%, 1.51%, 1.62%, and 1.47%, respectively. Cui Jing developed a high-sensitive triaxial tactile sensor based on the multilayer capacitor structure [13]. The maximum interference error between the shear X and Y directions and between the shear and normal directions are 7.636% and 1.051%, respectively. MA. A Pajhouh reported a three-dimensional force sensor based on a T-shaped elastic structure. The cross-interference error is effectively suppressed to 0.56% in the measuring range of ±50 N, but another one appears to be 12.37% [14]. Qiaokang Liang presented a six-dimensional wrist force/torque sensor based on E-type membranes compared to the conventional sensor based on cross beams, whose maximum interference error is 1.6% [15]. Xiu He proposed a novel three-dimensional force sensor based on an ultrasensitive all-fiber extrinsic Fabry–Perot strain sensor as well as a paperclip-shaped elastomer. Experimental results show that all of the independent coefficients are significantly larger than the corresponding interference coefficients; however, cross-interference errors were not quantified [8].

(1) $$E_{F_{y}\to F_{x}}=\frac{FSO_{F_{y}}}{FSO_{F_{x}}}\times 100\%,$$

Thereafter, researchers tried to use machine learning and decoupling algorithms in a three-dimensional force sensor, hoping to further suppress the cross-interference. For example, Shizheng Sun reported a fiber Bragg grating (FBG) three-dimensional force sensor based on the sparrow search algorithm-extreme learning machine. Research shows that the maximum cross-interference error of this decoupling method is 1.18% [16]. Yang Song presents an intelligent back-propagation decoupling algorithm for a flexible tactile sensor, and the experiment shows that the best average decoupling error is 1.69% [17].

Cross-interference suppression has always been a technical challenge in the design and manufacturing process of three-dimensional force sensors [18]. This paper presents another method for cross-interference suppression, by using the symmetrical stress distribution of elastic deformation element and the balance principle of Wheatstone bridge. According to the proposed method, two three-dimensional force sensors, based on octagonal ring structure and semi-conductive strain gauge, are fabricated for verification.

## 2. Principles and Methods

### 2.1. Elastic Deformation Element

**Figure 1:**
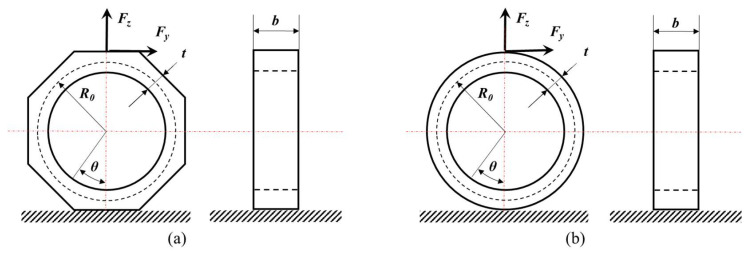
Structural diagram of thin ring. (**a**) thin-octagonal ring; (**b**) thin-circular ring.

The first step in three-dimensional force sensor design is to select an appropriate elastic deformation element [19], and the octagonal ring is commonly used, as shown in Figure 1a. In previous studies, the thin-circular ring (*t*/*R*_0_ ≤ 1/5) theory was often used to approximate the surface stress distribution of thin-octagonal ring. For the bottom-fixed thin-circular ring in Figure 1b, its surface normal stress caused by horizontal force *F_y_* and vertical force *F_z_* can be described in the following equations [9]:

(2) $$\sigma_{F_{y}}=\pm \frac{3F_{y}R_{0}\cos\theta }{bt^{2}},$$

(3) $$\sigma_{F_{z}}=\pm \frac{6F_{z}R_{0}}{bt^{2}}\left(\frac{1}{2}\sin\theta -\frac{1}{\pi }\right),$$

where *σ_Fy_* and *σ_Fz_* denote normal stress caused by *F_y_* and normal stress caused by *F_z_*, respectively; *R*_0_, *b*, and *t* refer to average radius, width, and thickness of the thin-circular ring; *θ* presents the location of any position on the thin-circular ring. Formulas (2) and (3) indicate that *F_y_* causes no stress at the position of *θ* = 90°, and *F_z_* causes no stress at the position of *θ* = arcsin (2/π) ≈ 39.54°. Positions where stress equals zero are called “strain node”, which can help in avoiding cross-interference. For example, strain gauge placed at *θ* = 90° is only sensitive to *F_z_* since *F_y_* causes no stress here. Similarly, strain gauge placed at *θ* = 39.54° is only sensitive to *F_y_* since *F_z_* causes no stress here.

For the octagonal ring, there are literatures reporting that good results are obtained when the strain gauges are located at the position of *θ* = 90° and *θ* = 45°, respectively [20,21].

However, it has some defects:

(1) Some research has pointed out that the value of *θ* (position of strain node) changes with the size variation of octagonal ring, which is not a fixed value [22,23]. 

(2) Strain node represents a line segment (which has no width) on the octagonal ring, while strain gauge has a length and a width. Specifically, the strain gauge bonded at a strain node will simultaneously be affected by both *F_y_* and *F_z_*, and thus cross-interference occurs.

Therefore, the strain node may not be a feasible solution for cross-interference suppression. In this paper, structure symmetry is adopted to solve this problem.

### 2.2. Finite Element Analysis of Octagonal Ring

**Figure 2:**
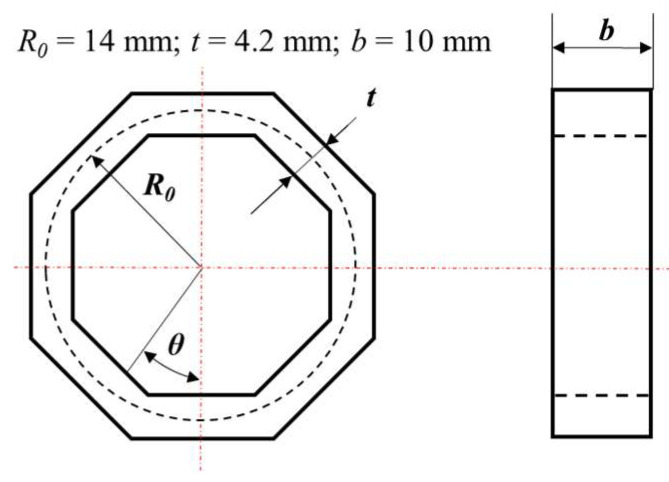
Structure and size parameters of the proposed octagonal ring.

Figure 2 depicts the modified octagonal ring that is used in this paper, which is contrary to the octagonal ring in Figure 1a, as both its inner and outer surfaces are octagonal rings.

**Figure 3:**
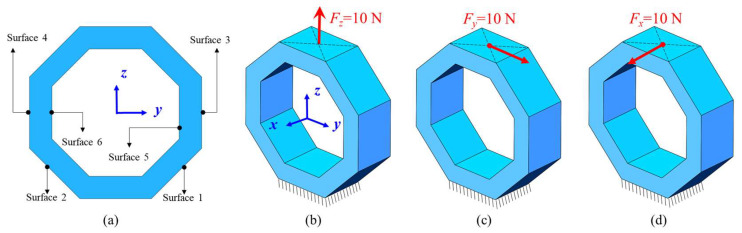
The physical model for finite element simulation: (**a**) Surface definition; (**b**) stress analysis of *F_z_*; (**c**) stress analysis of *F_y_*; (**d**) stress analysis of *F_x_*.

The finite element simulation is adopted for octagonal ring stress analysis. Figure 3 and Table 1 provide the physical model and parameter settings for finite element simulation. The octagonal ring is fixed on its bottom surface, and the stress distribution of surface 1~surface 6 is analyzed under *F_x_*, *F_y_*, and *F_z_*, respectively.

#### 2.2.1. Surface Stress Distribution of Octagonal Ring under *F_z_*

**Figure 4:**
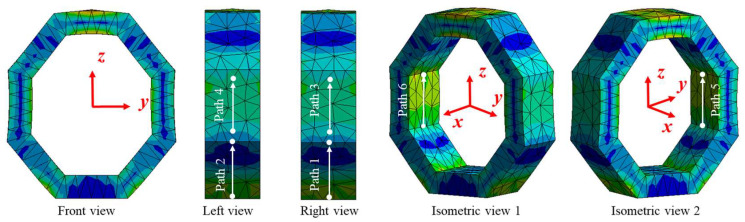
Von Mises stress distribution on the octagonal ring under *F_z_*.

**Figure 5:**
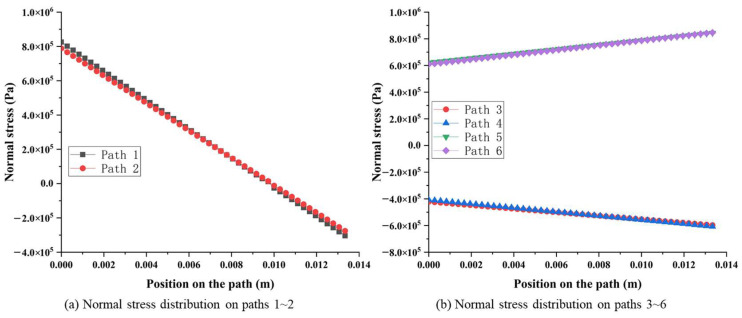
Normal stress distribution on pre-set paths of the octagonal ring under *F_z_*.

According to the simulation results, the stress distribution between surfaces 1 and 2, surfaces 3 and 4, as well as surfaces 5 and 6 are highly symmetrical, as shown in Figure 4. In order to provide a more detailed demonstration of the above symmetry characteristic, Figure 5 depicts the stress amplitude curves along the symmetric paths from surface 1 to surface 6, respectively. Considering surfaces 1 and 2 as an example, the normal stress on pre-set path 1 and path 2 changes from about 8.0 × 10^5^ Pa to −3.0 × 10^5^ Pa, and the stress on every corresponding position in path 1 and path 2 has the same value and sign as in Figure 5a. Similarly, normal stress on surface 3 and surface 4 changes from about −4.0 × 10^5^ Pa to −5.0 × 10^5^ Pa, with the same value and sign in symmetrical positions. Moreover, normal stress on surface 5 and surface 6 changes from about 6.0 × 10^5^ Pa to 8.0 × 10^5^ Pa, with the same value and sign in symmetrical positions, as shown in Figure 5b.

#### 2.2.2. Surface Stress Distribution of Octagonal Ring under *F_y_*

**Figure 6:**
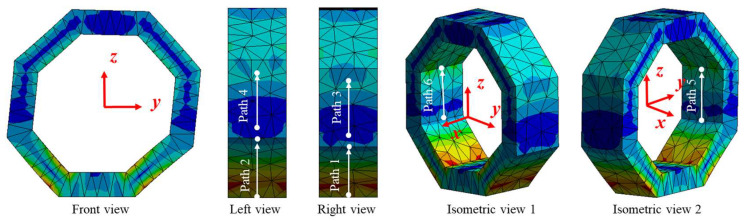
Von Mises stress distribution on the octagonal ring under *F_y_*.

**Figure 7:**
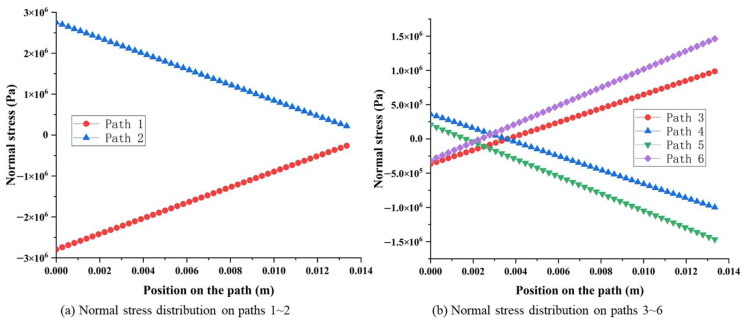
Normal stress distribution on pre-set paths of the octagonal ring under *F_y_*.

The stress distribution between surfaces 1 and 2, surfaces 3 and 4, as well as surfaces 5 and 6 are still highly symmetrical, as shown in Figure 6, and the difference is that the sign of the stress is opposite. Figure 7 depicts the stress amplitude curves along the symmetric paths from surface 1 to surface 6, respectively. Considering surfaces 1 and 2 as an example, the normal stress on path 1 and path 2 changes from about 2.75 × 10^5^ Pa to 0.25 × 10^5^ Pa (−2.75 × 10^5^ Pa to −0.25 × 10^5^ Pa), and the stress on every corresponding position in path 1 and path 2 is equal in value but opposite in sign, as shown in Figure 7a. Similarly, normal stress on surface 3 and surface 4 changes from about 3.60 × 10^5^ Pa to −9.90 × 10^5^ Pa (−3.60 × 10^5^ Pa to 9.90 × 10^5^ Pa), with the same value and opposite sign in symmetrical positions. Moreover, normal stress on surface 5 and surface 6 changes from about 2.00 × 10^5^ Pa to −1.50 × 10^6^ Pa (−2.00 × 10^5^ Pa to 1.50 × 10^6^ Pa), with the same value and opposite sign in symmetrical positions, as shown in Figure 7b.

#### 2.2.3. Surface Stress Distribution of Octagonal Ring under *F_x_*

**Figure 8:**
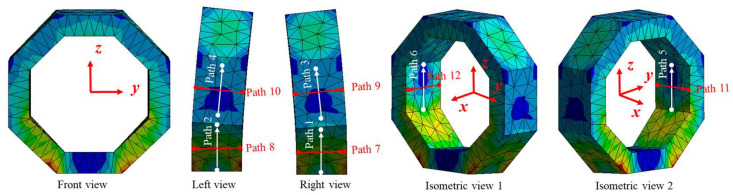
Von Mises stress distribution on the octagonal ring under *F_x_*.

**Figure 9:**
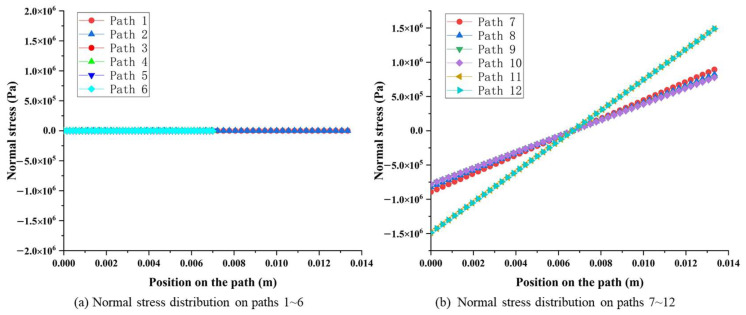
Normal stress distribution on pre-set paths of the octagonal ring under *F_x_*.

The stress distribution between surfaces 1 and 2, surfaces 3 and 4, as well as surfaces 5 and 6 show highly symmetrical characteristics, as shown in Figure 8. Moreover, the normal stress (on paths 1~6) caused by *F_x_* is almost zero when compared with the stress caused by *F_y_* and *F_z_*, which can be ignored, as shown in Figure 9a. 

Furthermore, the stress amplitude on each surface shows the characteristic of symmetry. Considering surface 1 and surface 2 as an example, the normal stress on paths 7 and 8 are symmetrically distributed along the vertical centerline, changing from −8.90 × 10^5^ Pa to 8.90 × 10^5^ Pa with the same value and opposite sign, as shown in Figure 9b. Similarly, the same stress distribution rules exist from surface 3 to surface 6.

In addition to the above finite element simulation method, mechanoluminescent technology is a useful method for studying the stress distribution of elastic element [24], which may assist in visualizing the stress distribution directly.

### 2.3. Cross-Interference Suppression Method

**Figure 10:**
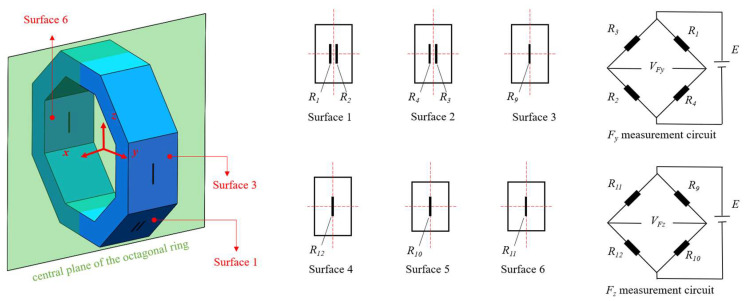
Strain gauge location and Wheatstone bridge arrangement for cross-interference suppression.

Wheatstone bridge is a typical measuring circuit for strain gauge sensors. To make the measuring circuit an anti-cross-interference, strain gauges *R*_1_~*R*_4_ and *R*_9_~*R*_12_ are arranged symmetrically on surface 1~surface 6, as shown in Figure 10, where *R*_1_ and *R*_2_ are symmetric to each other on surface 1, *R*_3_ and *R*_4_ are symmetric to each other on surface 2. Moreover, *R*_1_~*R*_4_ should be symmetric to each other at the central plane of the octagonal ring. Strain gauges *R*_9_~*R*_12_ are located at the center of surface 3~surface 6, with the assumption that all strain gauges have the same size, gauge factor (*GF*), and original resistance (*R*_0_). According to the stress distribution characteristic in Section 2.2, the resistance change in strain gauges caused by *F_x_*, *F_y_*, and *F_z_*, respectively is listed in Table 2.

For *F_y_* measurement circuit, the voltage output caused only by *F_y_* is presented in Equation (4), while the voltage output caused by *F_y_*, *F_z_*, and *F_x_* is presented in Equation (5).

(4) $$\begin{array}{ll} V_{Fy} & =\left(\frac{R_{4}}{R_{1}\;+\;R_{4}}-\frac{R_{2}}{R_{2}\;+\;R_{3}}\right)\cdot E\\ & =\left(\frac{R_{0}\;+\;\Delta r_{1}}{R_{0}\;-\;\Delta r_{1}\;+\;R_{0}\;+\;\Delta r_{1}}-\frac{R_{0}\;-\;\Delta r_{1}}{R_{0}\;-\;\Delta r_{1}\;+\;R_{0}\;+\;\Delta r_{1}}\right)\cdot E\\ & =\frac{\Delta r_{1}}{R_{0}}\cdot E \end{array},$$

(5) $$\begin{array}{ll} V_{Fy} & =\left(\frac{R_{4}}{R_{1}\;+\;R_{4}}-\frac{R_{2}}{R_{2}\;+\;R_{3}}\right)\cdot E\\ & =\left(\begin{array}{l} \frac{R_{0}\;+\;\Delta r_{1}\;+\;\Delta r_{3}\;+\;\Delta r_{5}}{R_{0}\;-\;\Delta r_{1}\;+\;\Delta r_{3}\;-\;\Delta r_{5}\;+\;R_{0}\;+\;\Delta r_{1}\;+\;\Delta r_{3}\;+\;\Delta r_{5}}-\\ \frac{R_{0}\;-\;\Delta r_{1}\;+\;\Delta r_{3}\;+\;\Delta r_{5}}{R_{0}\;-\;\Delta r_{1}\;+\;\Delta r_{3}\;+\;\Delta r_{5}\;+\;R_{0}\;+\;\Delta r_{1}\;+\;\Delta r_{3}\;-\;\Delta r_{5}} \end{array}\right)\cdot E\\ & =\frac{\Delta r_{1}}{R_{0}\;+\;\Delta r_{3}}\cdot E \end{array},$$

The difference between Equations (4) and (5) indicates that *F_z_* will interfere with *F_y_*’s measurement result, and the theoretical cross-interference error is:

(6) $$E=\frac{\frac{\Delta r_{1}}{R_{0}}\cdot E-\frac{\Delta r_{1}}{R_{0}\;+\;\Delta r_{3}}\cdot E}{\frac{\Delta r_{1}}{R_{0}}\cdot E}\times 100\%=\frac{\Delta r_{3}}{R_{0}+\Delta r_{3}}\times 100\%,$$

According to the principle of semiconductor piezoresistive effect [9]:

(7) $$\frac{\Delta r_{3}}{R_{0}}=GF\cdot \varepsilon ,$$

where *GF* is the gauge factor of the strain gauge (which is 150 for the semi-conductive strain gauge used in this paper), and *ε* is the strain at the strain gauge’s location. 

Finite element simulation results show that the maximum normal strain at the strain gauge bonding position is 7.5 × 10^−6^. Therefore, the maximum value of theoretical cross-interference error is 0.112% as shown below, which can be ignored.

(8) $$E=\frac{\Delta r_{3}}{R_{0}+\Delta r_{3}}\times 100\%=\frac{\frac{\Delta r_{3}}{R_{0}}}{1+\frac{\Delta r_{3}}{R_{0}}}\times 100\%=\frac{GF\cdot \varepsilon }{1+GF\cdot \varepsilon }\times 100\%=\frac{150\times 7.5\times 10^{-6}}{1+150\times 7.5\times 10^{-6}}=0.112\%,$$

For *F_z_* measurement circuit, the voltage output caused only by *F_z_* is the same as the output caused by *F_y_*, *F_z_*, and *F_x_*, as illustrated in Equations (9) and (10). This indicates that the *F_z_* measurement circuit can independently measure *F_z_* without being interfered by *F_y_* or *F_x_*.

(9) $$\begin{array}{ll} V_{Fz} & =\left(\frac{R_{10}}{R_{9}\;+\;R_{10}}-\frac{R_{12}}{R_{11}\;+\;R_{12}}\right)\cdot E\\ & =\left(\frac{R_{0}\;+\;\Delta r_{4}}{R_{0}\;-\;\Delta r_{4}\;+\;R_{0}\;+\;\Delta r_{4}}-\frac{R_{0}\;-\;\Delta r_{4}}{R_{0}\;+\;\Delta r_{4}\;+\;R_{0}\;-\;\Delta r_{4}}\right)\cdot E\\ & =\frac{\Delta r_{4}}{R_{0}}\cdot E \end{array},$$

(10) $$\begin{array}{ll} V_{Fz} & =\left(\frac{R_{10}}{R_{9}\;+\;R_{10}}-\frac{R_{12}}{R_{11}\;+\;R_{12}}\right)\cdot E\\ & =\left(\begin{array}{l} \frac{R_{0}\;-\;\Delta r_{2}\;+\;\Delta r_{4}}{R_{0}\;+\;\Delta r_{2}\;-\;\Delta r_{4}\;+\;R_{0}\;-\;\Delta r_{2}\;+\;\Delta r_{4}}-\\ \frac{R_{0}\;-\;\Delta r_{2}\;-\;\Delta r_{4}}{R_{0}\;+\;\Delta r_{2}\;+\;\Delta r_{4}\;+\;R_{0}\;-\;\Delta r_{2}\;-\;\Delta r_{4}} \end{array}\right)\cdot E\\ & =\frac{\Delta r_{4}}{R_{0}}\cdot E \end{array},$$

### 2.4. Sensor Design and Fabrication

**Figure 11:**
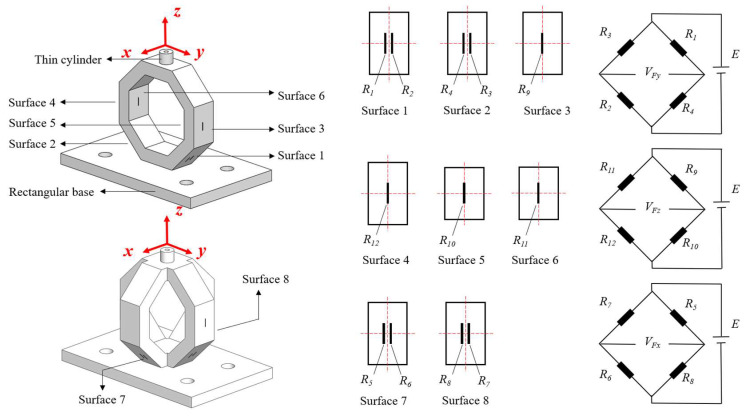
Three-dimensional force sensor and measuring circuit arrangement.

According to the above cross-interference suppression method, a type of three-dimensional force sensor is designed as shown in Figure 11. The sensor is composed of two mutually perpendicular octagonal rings, which can measure *F_y_*, *F_z_*, and *F_x_*. There is a rectangular base set at the bottom of the sensor, and a thin cylinder set at the top of the sensor for force loading. The dimensions of the sensor are shown in Table 3, and the measuring range is set as 0~20 N.

The strain gauge used in this paper is a semi-conductive strain gauge purchased from Anhui Tianguang sensor Co., Ltd. (Bengbu, China). The technical parameters of the strain gauge are listed in Table 4.

**Figure 12:**
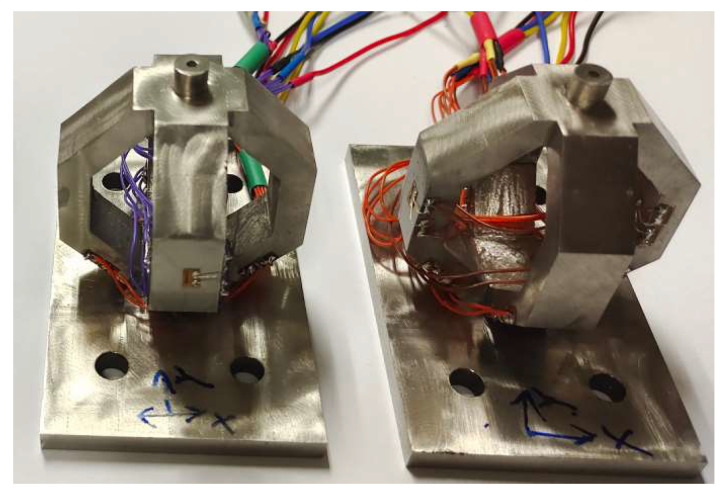
Photograph of the developed three-dimensional force sensors.

The sensor is fabricated by stainless steel 3D printing, and the semi-conductive strain gauges are bonded on the octagonal ring using M-Bond 610 glue produced from Vishay Micro-Measurements. The developed sensors are shown in Figure 12.

## 3. Test and Verification

### 3.1. Experiment Setup

**Figure 13:**
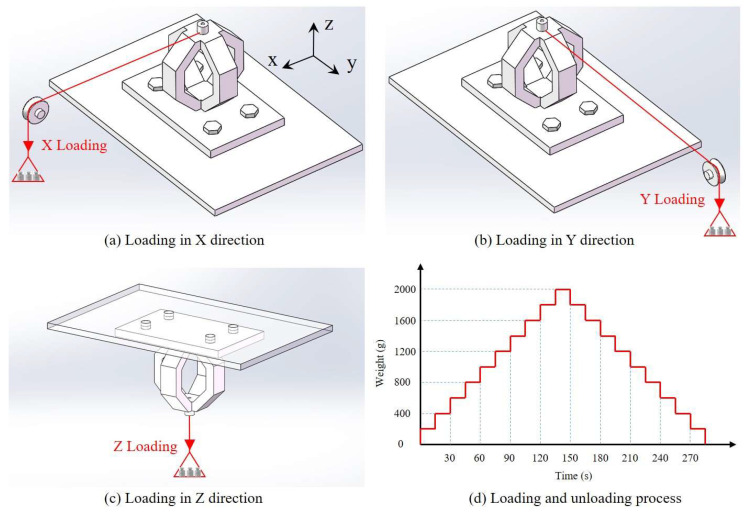
The schematic diaphragm of sensor calibration in X, Y, and Z directions.

To verify the feasibility of the proposed cross-interference suppression method, the calibration experiment is carried out as follows:

The sensor was fixed on a horizontal platform, and then the standard force was loaded by weight in X, Y, and Z directions, respectively, as shown in Figure 13. In each calibration cycle, the weight rises from 0 to 2000 g with an interval of 200 g, and then decreases from 2000 to 0 g.

The sensor is powered by a GPS-3303C power supplier with 5V DC, and the output signals are recorded by three Fluke-8846A high-precision digital multimeters. Calibration in each direction was performed at least three times and the measured results are averaged.

### 3.2. Results and Discussion

**Figure 14:**
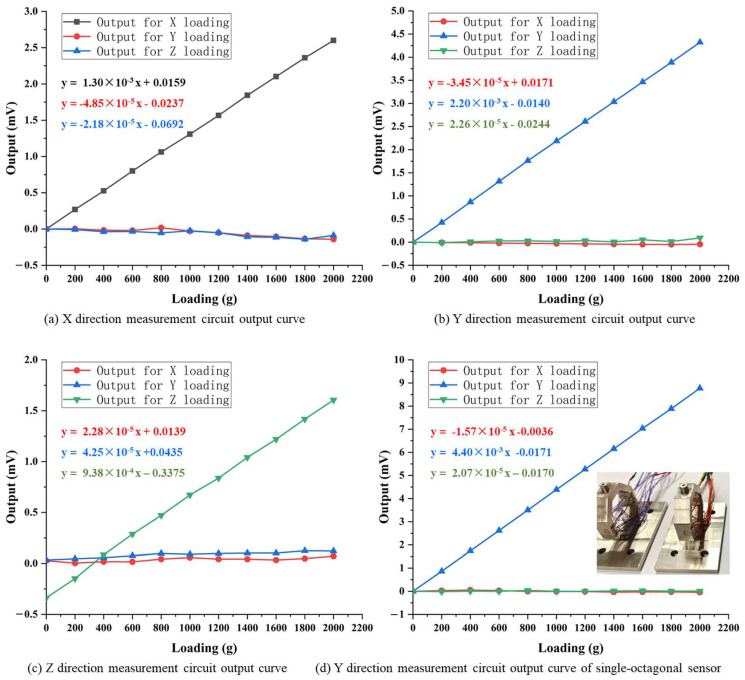
Measurement circuits’ output curves of static calibration.

Figure 14a–c depict the static calibration results of each measurement circuit under the action of loads in the X, Y, and Z directions, respectively. In Figure 14a, the *F_x_* measurement circuit exhibits good linear output characteristics under *F_x_*, and the slope of its fitting curve (i.e., output sensitivity) is 1.30 × 10^−3^ mV/g. The outputs of *F_x_* measurement circuit under the load in Y and Z directions are shown in red and blue curves, with slopes of −4.85 × 10^−5^ mV/g and −2.18 × 10^−5^ mV/g for their linear fitting curves. The output sensitivity caused by the load in Y and Z directions is nearly two orders of magnitude lower than that caused by the load in X direction.

The cross-interference errors of *F_x_* measurement circuit under Y and Z direction loads can be calculated by Formula (1). Considering the random error in static calibration results, this paper uses the output sensitivity (i.e., the slope of the fitting curve) to replace the full-scale output of the sensor for cross-interference error calculation, as shown in Formula (11):

(11) $$E_{F_{y}\to F_{x}}=\frac{S_{F_{y}}}{S_{F_{x}}}\times 100\%,$$

where *S_Fy_* and *S_Fx_* are the output sensitivity of *F_x_* measurement circuit during Y and X direction calibration, respectively.

The cross-interference errors of *F_x_* measurement circuit under Y and Z direction loads are −3.73% and −1.68%, respectively, as shown in Table 5, which indicates that the load in Y and Z directions has little impact on the output of *F_x_* measurement circuit. This proves that the *F_x_* measurement circuit designed using the principle of stress symmetry distribution and Wheatstone bridge balance principle has good anti-cross-interference ability.

In Figure 14b, the *F_y_* measurement circuit also exhibits good linear output characteristics under *F_y_*, and the slope of its fitting curve (i.e., output sensitivity) is 2.20 × 10^−3^ mV/g. The outputs of *F_y_* measurement circuit under the load in X and Z directions are shown in red and green curves, with slopes of −3.45 × 10^−5^ mV/g and 2.26 × 10^−5^ mV/g for their linear fitting curves. The output sensitivity caused by the load in X and Z directions is nearly two orders of magnitude lower than that caused by the load in Y direction. The cross-interference errors of *F_y_* measurement circuit under X and Z direction loads are −1.57% and 1.03%, respectively. This indicates that the load in X and Z directions has little impact on the output of *F_y_* measurement circuit, which means that the *F_y_* measurement circuit designed using the principle of stress symmetry distribution and Wheatstone bridge balance principle has good anti-cross-interference ability, as well.

In Figure 14c, the slope (i.e., output sensitivity) of *F_z_* measurement circuit under Z direction load is 0.938 × 10^−3^ mV/g. The output sensitivities of *F_z_* measurement circuit under X and Y direction loads are −2.28 × 10^−5^ mV/g and 4.25 × 10^−5^ mV/g, respectively. According to Formula (11), the cross-interference errors of *F_z_* measurement circuit under X and Y direction loads are 2.43% and 4.53%, respectively, which is also nearly two orders of magnitude lower than that caused by the load in Z direction.

Based on the static calibration and cross-interference error results of *F_x_*, *F_y_*, and *F_z_* measurement circuits, the following points can be drawn:

(1) The *F_x_* and *F_y_* measurement circuits have good anti-cross-interference ability, especially *F_y_* measurement circuit, which has a lower cross-interference error. However, the *F_x_* and *F_y_* measurement circuits are identical in terms of elastic element structure, strain gauge placement, and measurement circuit organization; therefore, their cross-interference error should also be the same. Moreover, the measured cross-interference errors are higher than the theoretical calculated results by Formula (8).

This is mainly due to the fact that during sensor design and cross-interference theoretical calculation, the preconditions are ideal, such as the initial resistance and gauge factor of all strain gauges are completely equal, and the distribution of all strain gauges are completely symmetrical. However, in practical packaging process, it is impossible to ensure that all the strain gauges in each measuring circuit have the same initial resistance and gauge factor, and the strain gauges’ position error and parallelism deviation are inevitable. These may cause inconsistent resistance change in strain gauges in the measuring circuit, and cause unwanted output when the measuring circuit is subjected to loads in crossing directions. The solution to this problem is to optimize the packaging process of strain gauge, improve the consistency of the initial resistance and gauge factor of the strain gauges, and reduce the position error and parallelism deviation of the strain gauges.

To verify the rationality of the above analysis, four strain gauges with highly similar initial resistance and gauge factor are packaged on an octagonal ring to form a *F_y_* measuring circuit, and the position error and parallelism deviation between the strain gauges are strictly controlled. The cross-interference errors of “*F_x_→F_y_*” and “*F_z_→F_y_*” calculated from the static calibration are −0.36% and 0.47%, respectively, as shown in Figure 14d. This indicates that the cross-interference error can be effectively reduced by improving the packaging technology.

(2) Although *F_z_* measurement circuit exhibits good ability in cross-interference suppression, its maximum cross-interference error is higher than *F_x_* and *F_y_* measurement circuits, which is inconsistent with the conclusion in Section 2.3 that the theoretical cross-interference error is zero.

This is mainly due to the fact that in elastic element design, it is assumed that the stress amplitudes are equal at corresponding positions from surface 3 to surface 6. In fact, the stress is not strictly equal at corresponding positions, as shown in Figure 5b, Figure 7b and Figure 9b, which makes the anti-cross-interference ability of *F_z_* measurement circuits inferior to the *F_x_* and *F_y_* measurement circuits.

For *F_z_* measurement circuit, the cross-interference error caused by *F_y_* is higher than that caused by *F_x_*. This is due to the fact that in X direction loading, all strain gauges in *F_z_* measurement circuit are located on the neutral layer of each surface, and the stress generated on the strain gauges is quite small as shown in Figure 9a, resulting in a relatively small output under the action of *F_x_*. In Y direction loading, the stress generated on the strain gauges is non-negligible and inconsistent as shown in Figure 7b, resulting in unwanted output and making the *F_z_* measurement circuit more susceptible to cross-interference from *F_y_*.

To further reduce the cross-interference error of *F_z_* measurement circuit, a direct method is to make the stress generated on surfaces 3~6 equal to the greatest extent possible. The solution is through the use of a thinner octagonal ring, since for the thin structure, the stress on its inner surface and outer surface can easily be equal. For the thin octagonal ring, it is beneficial for improving the stress amplitude at the strain gauge location, which helps in improving the output sensitivity of *F_z_* measurement circuit and reducing its cross-interference error. However, using the thin octagonal ring will reduce the load-bearing capacity in the X and Y directions, which needs to be considered during sensor design.

Table 6 presents a comparison of cross-interference errors between this paper and other research. The developed three-dimensional force sensor in this paper demonstrates good anti-cross-interference ability, which proves the feasibility of the cross-interference suppression method proposed.

## 4. Conclusions

Aiming at the cross-interference suppression in three-dimensional force measurement, this paper proposes a three-dimensional force sensor design method based on the symmetrical stress distribution and the balance principle of Wheatstone bridge. The experimental results demonstrate the following:

(1) The proposed sensor design method is experimentally verified to be feasible, and the maximum and minimum cross-interference errors of the developed sensor are 4.53% and 1.03%, respectively. Moreover, research shows that by improving the consistency of the initial resistance and gauge factor of strain gauges, as well as reducing the position error and parallelism deviation of strain gauges, this can further reduce the cross-interference error.

(2) The output sensitivity of *F_z_* measurement circuit is only 72.15% and 42.64% of *F_x_* and *F_y_* measurement circuits. Using a thinner octagonal ring can effectively improve the output sensitivity and the consistency of strain gauge resistance changes in *F_z_* measurement circuit, thereby reducing the cross-interference error.

Future work will focus on optimizing the packaging technology of strain gauges and the thickness of octagonal ring to further reduce the cross-interference error.

## Figures and Tables

**Table 1:** Parameter settings for finite element simulation.

**Material**	**Density**	**Tensile Yield Strength**	**Compressive Yield Strength**	**Bulk Modulus**
Structural steel	7852 kg/m^3^	2.5 × 10^8^ Pa	2.5 × 10^8^ Pa	1.6667 × 10^11^ Pa
**Shear Modulus**	**Young’s Modulus**	**Tensile Ultimate Strength**	**Compressive Ultimate Strength**	**Poisson’s Ratio**
7.692 × 10^10^ Pa	2.0 × 10^11^ Pa	4.6 × 10^8^ Pa	0 Pa	0.3

**Table 2:** Resistance change in strain gauges caused by *F_x_*, *F_y_*, and *F_z_*.

Force	Resistance Variation							
	*R*_1_	*R*_2_	*R*_3_	*R*_4_	*R*_9_	*R*_10_	*R*_11_	*R*_12_
*F_y_*	−Δ*r*_1_	−Δ*r*_1_	+Δ*r*_1_	+Δ*r*_1_	+Δ*r*_2_	−Δ*r*_2_	+Δ*r*_2_	−Δ*r*_2_
*F_z_*	+Δ*r*_3_	+Δ*r*_3_	+Δ*r*_3_	+Δ*r*_3_	−Δ*r*_4_	+Δ*r*_4_	+Δ*r*_4_	−Δ*r*_4_
*F_x_*	−Δ*r*_5_	+Δ*r*_5_	−Δ*r*_5_	+Δ*r*_5_	0	0	0	0

**Table 3:** Main dimensions of the developed three-dimensional force sensor.

Thin Cylinder		Octagonal Ring			Rectangular Base	
Diameter	Height	*R*_0_	*t*	*b*	Size	Circular hole
6 mm	5 mm	14 mm	4.2 mm	10 mm	70 × 40 × 4 mm^3^	Φ 7 mm

**Table 4:** Technical parameters of the semi-conductive strain gauge.

Resistance	Gauge Factor	Resistor Size	Base Size	Strain Limitation	Temperature
1000 Ω	150 ± 5%	3.8 × 0.22 mm^2^	5.0 × 3.0 mm^2^	3000 με	<80 °C

**Table 5:** Cross-interference errors summarized from sensor calibration results.

Cross-Interference Error	*F_y_*→*F_x_*	*F_z_*→*F_x_*	*F_x_*→*F_y_*	*F_z_*→*F_y_*	*F_x_*→*F_z_*	*F_y_*→*F_z_*
3D force sensor	−3.73%	−1.68%	−1.57%	1.03%	2.43%	4.53%

**Table 6:** Cross-interference errors comparison between different literatures.

Literatures		[9]	[10]	[11]	[12]	[13]	[14]	This Paper
Cross-interference error	Maximum	3%	25% *	40% *	2.41%	7.64%	12.37%	4.53%
	Minimum	1%	4% *	/	1.47%	1.05%	0.56%	1.03%

* indicates that the cross-interference error is estimated from the figure of the literature.

## Data Availability

The data presented in this study are available on request from the corresponding author. The data are not publicly available due to privacy.
